# Weismann-Netter-Stuhl Syndrome:A family report

**DOI:** 10.4274/jcrpe.v1i4.45

**Published:** 2010-12-08

**Authors:** Hayrullah Alp, Mehmet Emre Atabek, Özgür Pirgon

**Affiliations:** 1 Department of Pediatrics, Selcuk University, Meram Medical Faculty, Konya; 2 Department of Pediatric Endocrinology, Selcuk University, Meram Medical Faculty, Konya; 3 Department of Pediatric Endocrinology, Konya Research and Training Hospital, Konya; +90-332-223 63 10+90-332-223 61 82drhayrullahalp@hotmail.comSelcuk University, Meram Medical Faculty, Department of Pediatrics Beysehir Yolu 42080-Konya, Turkey

**Keywords:** Weismann-Netter-Stuhl syndrome, femur involvement, radiography

## Abstract

Weismann-Netter-Stuhl (WNS) syndrome is a rare skeletal anomaly that affects the diaphyseal part of both the tibiae and fibulae with posterior cortical thickening and anteroposterior bowing. This anomaly is usually bilateral and symmetrical. The patients are generally of short stature. In some cases, a family history suggesting genetic transmission of a mutation with an unknown locus has been reported. In this paper we present an infant with WNS syndrome with bilateral involvement of the femur. Similar clinical findings were defined in three other family members.

**Conflict of interest:**None declared.

## INTRODUCTION

Weismann-Netter-Stuhl (WNS) syndrome is a rare diaphyseal dysplasia that was first described in 1954 by Weismann- Netter and Stuhl.^1^ The primary clinical features are bowing of the lower extremities and short stature. Characteristic radiographic findings of the syndrome are bilateral anterior bowing of both tibiae and fibulae with posterior thickening.^1, 2^ Thickening and enlargement of the fibulae are described as tibialisation and confirm the diagnosis. Laboratory investigations reveal no abnormalities, and this is a feature which distinguishes this syndrome from rickets. Occasional bone lesions seen in WNS syndrome are femoral incurvature and exostoses, bilateral coxa vara, bowing of radius and ulna, metacarpal shortening, dolichophalangy, kyphoscoliosis, costal deformity, iliac wings and horizontal sacrum.^3, 4, 5, 6, 7, 8^ In this article we report an infant diagnosed with WNS syndrome. Clinical and radiographic findings suggestive of the syndrome were identified in three other family members.

## CASE REPORTS

**Figure 1:**
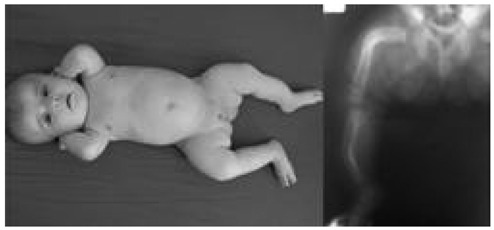
Antero-posterior bowing of both tibiae and fibulae with lateral bowing of the femurs.

**Figure 2:**
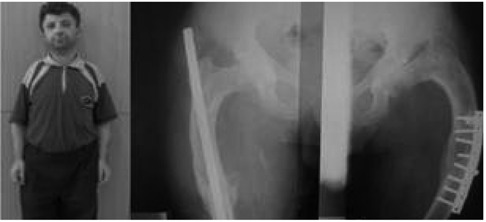
Short stature and palatine implanted femurs with lateral bowing.

A 3-month-old girl was referred to our clinic because of short stature and bowing of the legs. She was born at term as the second child of the family. The first child was a girl and had no health problems. On physical examination her length was 58 cm (SDS –1.75) and weight was 4200 gr (SDS –1.02). Physical examination revealed anterior external bowing of both legs which was more pronounced on the left side (Figure 1). Other clinical findings and routine laboratory assessments including serum phosphorus, calcium and alkaline phosphate levels were normal. Radiographic examination of the legs revealed bilateral incurvature of the femurs and anterior-internal bowing of both tibia and fibulae (Figure 1). Thickening of fibulae was detected bilaterally. The family history revealed that her father (height SDS –3.6) was operated on both femurs because of bilateral incurvature and platinum was emplaced two years earlier (Figure 2). In spite of this intervention, the father was short and bowing of both tibia and fibulae were apparent. The grandmother (height SDS –3.2) and an uncle (height SDS –3.4) also had bilateral bowing of the lower extremities. Laboratory evaluations were normal in these family members. The patient was diagnosed as WNS syndrome based on radiological findings and at present, the patient’s follow-up at our clinic is ongoing.

## DISCUSSION

WNS syndrome is known as tibioperoneal diaphyseal toxopachyosteosis and characterized by short stature and bowing of both tibiae and fibulae. Most of the reported cases, particularly in adult patients, have been published in French.^5^ Case reports of WNS in children are rare and its diagnosis is usually delayed in this age group.^3, 9^ The reareason for this delay may be due to unclear clinical symptoms and unfamiliar radiological appearance of the syndrome.

Increased diameter and thickening and enlargement of the fibulae is defined as tibialization, which is the principle finding that confirms the diagnosis of WNS syndrome. The involvement is frequently bilateral, and symmetrical.^2, 3, 4, 5, 6^ Unilateral involvement is very rare.^5^ Bowing of tibiae and fibulae is apparent at antero-posterior position on roentgenograms. Apex of bowing is classically at the junction of the upper two-thirds and lower third of the diaphysis.^10, 11^ Tibial exostosis has been reported in some patients.^6^ However, upper extremities and femurs may also be involved.^9, 10^ Another major finding of the syndrome is short stature. The reported heights of adult cases are within the range of 135-155 cm, the majority being between 145-155 cm.^5^

The patient in this report was diagnosed as WNS syndrome, based on the radiographic findings. The main clinical features that caused the family to seek medical opinion were bowing of the legs and short stature. On roentgenogram, bilateral antero-posterior and lateral bowing of tibiae and fibulae with involvement of femur, confirmed the diagnosis. Femur involvement is rarely reported in the literature. The family history revealed that the patient’s father and two other members of the family (i.e. the grandmother and uncle) were also affected.

Family history has not been commonly reported. So far, no genetic locus related to WNS syndrome has been detected. Siblings are rarely affected.^12^

In differential diagnosis of WNS syndrome, rickets should be taken into consideration. ^5^ Rickets can be excluded by biochemical testing.

In conclusion, WNS syndrome is a diagnostic entity, which may often be difficult to recognize. Especially in children, delayed or misdiagnosis is common. In some cases, family history can be helpful. Biochemical investigations can be useful in distinguishing WNS syndrome from rickets or other metabolic disorders.
